# LncRNAs and the Angiogenic Switch in Cancer: Clinical Significance and Therapeutic Opportunities

**DOI:** 10.3390/genes13010152

**Published:** 2022-01-15

**Authors:** Peace Mabeta, Rodney Hull, Zodwa Dlamini

**Affiliations:** 1 Angiogenesis Laboratory, Department of Physiology, Faculty of Health Sciences, University of Pretoria, Hatfield 0028, South Africa; 2 SAMRC Precision Oncology Research Unit (PORU), Pan African Cancer Research Institute (PACRI), University of Pretoria, Hatfield 0028, South Africa; rodney.hull@up.ac.za

**Keywords:** vascular endothelial growth factor, metastasis-associated lung adeno-carcinoma transcript 1, HOX antisense intergenic RNA, maternally expressed gene3, MANTIS, myocardial infarction associated transcript

## Abstract

Angiogenesis is one of the hallmarks of cancer, and the establishment of new blood vessels is vital to allow for a tumour to grow beyond 1–2 mm in size. The angiogenic switch is the term given to the point where the number or activity of the pro-angiogenic factors exceeds that of the anti-angiogenic factors, resulting in the angiogenic process proceeding, giving rise to new blood vessels accompanied by increased tumour growth, metastasis, and potential drug resistance. Long noncoding ribonucleic acids (lncRNAs) have been found to play a role in the angiogenic switch by regulating gene expression, transcription, translation, and post translation modification. In this regard they play both anti-angiogenic and pro-angiogenic roles. The expression levels of the pro-angiogenic lncRNAs have been found to correlate with patient survival. These lncRNAs are also potential drug targets for the development of therapies that will inhibit or modify tumour angiogenesis. Here we review the roles of lncRNAs in regulating the angiogenic switch. We cover specific examples of both pro and anti-angiogenic lncRNAs and discuss their potential use as both prognostic biomarkers and targets for the development of future therapies.

## 1. Introduction

Over the years the focus of cancer treatment has largely been on eliminating neoplastic cells. However, research has shown that most tumours need to establish a vascular supply through processes such as angiogenesis in order to grow beyond a critical size of 1–2 mm [1,2]. Angiogenesis is a complex multi-step process through which blood vessels are formed from a pre-existing microvasculature. The tumour vasculature can promote cancer progression, promote drug loss due to extravasation (leakage from a blood vessel) at the tumour site as well as drug resistance [2,3]. Angiogenesis involves a dynamic balance between pro- and anti-angiogenic factors which when disturbed can promote the development of various diseases [3]. The angiogenic switch, which represents the transition from the avascular tumour to the angiogenic phenotype, is driven by this shift in the balance between pro- and anti- angiogenic factors. Importantly, recent findings show that some of the factors that promote an angiogenic switch are regulated by noncoding ribonucleic acids [4,5,6]. Historically, deoxy-ribonucleic acid (DNA) that does not code for proteins was considered to possess no physiological relevance and was termed “junk DNA”. The human genome project has revealed that the amount of noncoding DNA exceeds that of coding DNA, with only about 2% of the genome coding for protein [7]. Further analysis of data from genomic platforms revealed that long non-coding RNA(LncRNA) accounted for approximately 68% of the human transcriptome [8]. LncRNAs are a major class of noncoding RNAs, are more than 200 base pairs (bps) in length and are tissue and cell specific. However, even within cells the transcripts tend to be compartment specific. Although lncRNAs are largely noncoding, there is a growing appreciation for their contribution to physiological function, and this also extends to the vasculature [4,5]. Recent investigations have shown that they play a role in orchestrating angiogenesis through gene regulation, both at the transcriptional and post-transcriptional levels, although much remains to be done to elucidate their mechanisms of action [6,7,8,9,10]. Research has further revealed the importance of lncRNAs in the maintenance of vascular homeostasis. LncRNAs, which regulate the process of blood vessel formation include myocardial infarction associated transcript (MIAT), which functions as a miR-150-5p sponge to regulate vascular endothelial growth factor (VEGF) expression [6,8,9,10]. Its inhibition results in decreased endothelial cell (EC) proliferation. In addition, MIAT silencing leads to reduced EC migration and tube formation [6,8]. Metastasis-associated lung adeno-carcinoma transcript 1 (MALAT1), which is required for the regulation of cell cycle proteins, is also an important regulator of physiological angiogenesis [7,8,9]. MALAT1 plays a crucial role in the adaptation of the vasculature to hypoxia. It is thus not surprising that angiogenesis is dysregulated in MALAT1 null mice [8,9]. GATA Binding Protein 6 antisense (GATA6-AS) is expressed by ECs and through its interaction with Lysyl oxidase-like (LOXL)2 promotes angiogenesis. It is also upregulated during hypoxia [10]. Spliced transcript—endothelial-enriched lncRNA (STEEL) regulates physiological angiogenesis by transcriptionally reducing the expression of endothelial nitric oxide synthase (eNOS) and other EC function modulators such as Kruppel-like Factor 2 (KLF2) [10]. Another lncRNA, MANTIS, also known as lncRNA n342419, regulates vascularisation mainly in response to changes in blood flow patterns [8,9]. The silencing of MALAT1 through siRNA reduces angiogenesis both in vitro and in vivo [7,8,9]. In contrast, non-coding repressor of NFAT (NRON) is a negative regulator of angiogenesis [10]. When dysregulated, lncRNAs promote pathophysiological states. Moreover, aberrant expression of lncRNAs in endothelial cells is observed in various diseases, including cancer [5,7].

## 2. Expression Patterns of lncRNAs in Endothelial Cells

**Figure 1:**
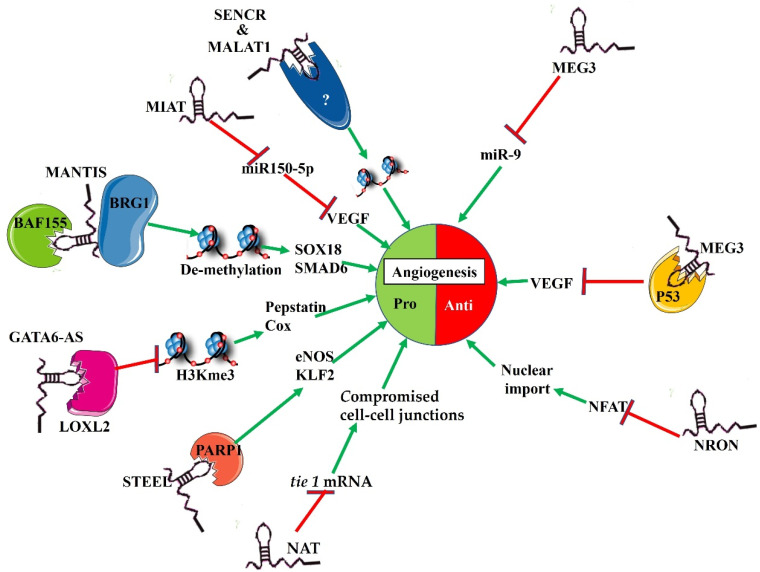
Pro- and anti-angiogenic lncRNAs. Pro-angiogenic lncRNAs such as MIAT bind to miRNA and interfere with the ability of these molecules to perform their function. LncRNAs such as NAT interfere with miRNA translation. GATA6-AS, MANTIS, MALAT1 and SENCR affect gene expression by altering methylation of target DNA. LncRNAs such as STEEL regulate the activity of transcription factors. Anti-angiogenic lncRNAs function by inhibiting the activity of molecules that stimulate angiogenesis.

Deep sequencing results have shown that approximately 56% of the total RNA in endothelial cells (ECs) is noncoding and mainly constituted by lncRNAs [7]. Generally, lncRNAs are expressed at low levels in the normal physiological setting [6,7]. However, they are upregulated in most neoplasms, although in a few instances they have been shown to be downregulated [5,6,10]. Among the first lncRNAs to be identified in ECs was nitric oxide synthase 3 antisense (NOS3AS), also known as autophagy 9-like 2 (APG9L2) [10]. The expression of NOS3AS correlates with low levels of the enzyme endothelial nitric oxide synthase (eNOS) [10]. LncRNA subtypes in endothelial cells include natural antisense transcripts (NATs), which constitutes about 7% of the total noncoding RNA identified in human umbilical vein endothelial cells (HUVECs) [11]. In ECs, the lncRNA NAT for tyrosine kinase with immunoglobulin-like and epidermal growth factor (EGF)- like domains 1 (TIE1) binds to TIE1 mRNA and decreases its transcript levels (Figure 1) [11,12]. This in turn results in compromised cell–cell junctions between ECs [11]. It is noteworthy that TIE is exclusively expressed in ECs and is upregulated in the tumour vasculature. LNC00323-003 and MIR503HG, which are both expressed by venous and arterial endothelial cells, are not compartment-restricted and occur in the cytoplasm as well as the nucleus [12]. The expression of these two lncRNAs in ECs is altered when there is oxygen deprivation. Additionally, LNC00323 promotes angiogenesis in vitro in hypoxic conditions. The reported findings may underscore the importance of these oxygen sensitive lncRNAs in tumour angiogenesis as this process is largely triggered by hypoxia. One of the highly expressed lncRNAs in ECs, MALAT1, functions to protect ECs from the effects of oxygen deprivation and nutrient deprivation by stimulating moderate autophagy [13]. MALAT1 is active during the initiation stage of autophagy and during autolysosomal fusion [13,14]. Autophagy is a process of cellular degradation in response to various stresses such as nutrient deprivation and it plays an important role in the maintenance of homeostasis. The process of autophagy occurs in stages, which include initiation, phagophore nucleation, autophagosome structure formation, and autolysosomal fusion that leads to the degradation of unwanted cellular components. In addition to MALAT1, angiogenic lncRNAs regulate EC autophagy at different stages. Maternally expressed gene (MEG3) and H19 regulate the initiation stage while highly upregulated in liver cancer (HULC) is involved in the elongation stage [14]. Autophagy enables stress tolerance in ECs, especially in the context of hypoxia or nutrient deprivation. The induction of autophagy also increases endothelial nitric oxide synthase expression and supports angiogenesis. Interestingly, when dysregulated, autophagy contributes to endothelial dysfunction and impaired angiogenesis [14,15]. In addition to increasing the stress tolerance of ECs, MALAT1 upregulates VEGF and angiopoietins (ANG) in the microvasculature and suppresses EC apoptosis [16]. Other important lncRNAs expressed in ECs are Linc00493 and MEG3 (Figure 1). Studies show that MEG3 is upregulated during senescence in late passage versus early passage ECs and contributes to the endothelial dysfunction associated with aging cells [17,18]. MEG3 promotes anti-angiogenesis through the suppression of miR-9 and VEGF [10,17,18]. LincRNA-ST8SIA3, also known as regulator of reprogramming (ROR), is a long, noncoding RNA located at 18q21.31 of chromatin that was detected in ECs and appears to promote angiogenesis [18]. In stem cells, ROR regulates self-renewal by modulating the functions of Oct4, Sox2, and Nanog. In endothelial cells, ROR regulates the proangiogenic VEGF. Importantly, VEGF is indispensable in the onset of the angiogenic switch [1,2]. Of note is that the silencing of ROR in vitro results in the downregulation of VEGF [16]. Several lncRNAs were reportedly expressed in ECs under conditions of hypoxia, including MEG8 and 9, as well as H19 [13,19]. Interestingly, the knockdown of H19 reduced the ability of ECs to form cords in an in-vitro assay of angiogenesis. MIR20HG and MIR22HG are also expressed by ECs subjected to hypoxic conditions [17]. Ubiquitin-conjugating enzyme E2C pseudogene 3 (UB32CP3) is a lncRNA that promotes epithelial to mesenchymal transition (EMT) and metastasis and has also been detected in ECs [4,19]. In models of co-cultured ECs and (hepatocellular carcinomas) HCC cells overexpressing UB32CP3, the ECs were stimulated to proliferate more rapidly [19]. Additionally, cell migration was increased markedly compared to controls that did not express UB32CP3 [19]. UB32CP3 has also been shown to induce an increase in microvessel density in vivo [3]. Hypoxia is the most important trigger of angiogenesis and understanding how these lncRNAs regulate EC behavior during hypoxic conditions, and how they affect angiogenesis in the tumour setting has clinical relevance.

## 3. Functional Mechanisms of lncRNAs in Angiogenesis

**Figure 2:**
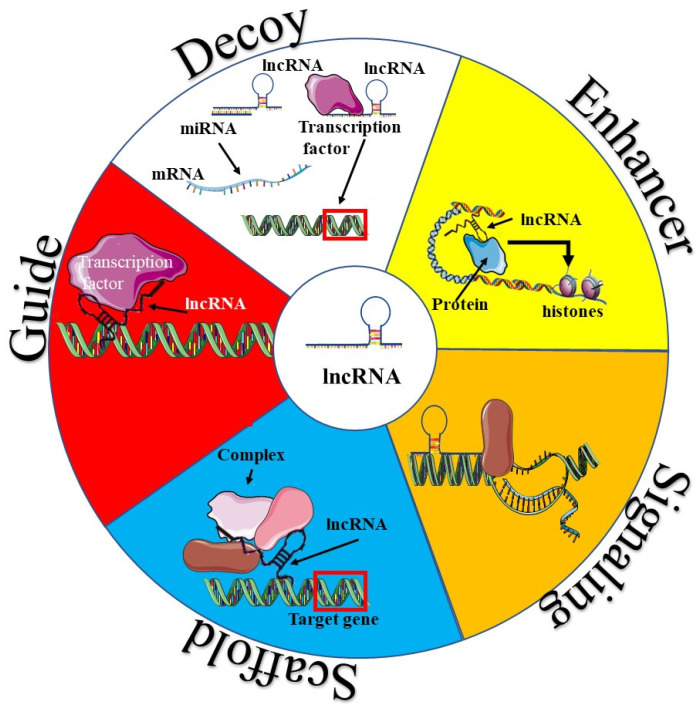
Mechanism of lncRNA action. Scaffold lncRNAs act as a framework for molecules such as proteins to bind to and be brought into close contact with each other, allowing them to perform their functions more easily. The red frame indicates the target sequence on the DNA strand. Guide lncRNAs recruit molecules such as proteins to a particular site on a nucleic acid molecule. Decoy lncRNAs act as decoy binding sites for molecules such as miRNAs or transcription factors. As such they are also known as sponge lncRNAs. Enhancer lncRNAs act to enhance the function of transcription factor-like molecule. Signalling lncRNAs act as signals to promote or repress the activity of transcription factors.

Long noncoding RNAs were classically divided into four archetypes based on their mechanism of action, namely, signalling, decoy, guide, and scaffold lncRNAs (Figure 2) [10,19,20]. Signalling lncRNAs regulate transcription by acting as molecular signals [6]. Decoy lncRNAs, on the other hand present alternate binding sites to catalytic and regulatory molecules, which include transcription factors and miRNAs [21,22]. This in turn limits the availability of these molecules and reduces their ability to modulate transcription. Guide lncRNAs support genomic positioning or localization, while scaffold lncRNAs provide a structural scaffold to enable the proper assembly of protein complexes such as ribonucleoproteins (RNP) [23,24,25]. Depending on the nature of the complex formed, it can induce transcriptional activation or suppression [25,26]. More recently, a fifth archetype, the enhancer lncRNA (eLncRNA) was described [6]. Enhancer lncRNAs stabilize and maintain chromatin loops [6,10].

The various lncRNA archetypes play important roles in the tumour vasculature by modulating gene expression at various levels. At the epigenetic level, lncRNAs recruit several epigenetic factors and regulate chromatin remodelling and gene splicing [27,28]. Plasmacytoma variant translocation 1 (PVT1) and LINC00313 are examples of angiogenesis regulating lncRNAs which exert their action at the epigenetic level [28]. Both PVT1 and LINC00313 combine with PRC2 and inhibit the transcription of ANGPTL4 and cell migration-regulating genes. ANGPTL4 regulates glucose metabolism in ECs and preserves the integrity of these cells [29]. Not surprising given its role in the regulation of EC function, ANGPTL4 also regulates angiogenesis [29]. Another long noncoding RNA in this category is H-19, which regulates angiogenesis in the tumour microvasculature and is upregulated in multiple cancers [28]. At the transcriptional level, lncRNAs regulate transcription through interactions with transcription factors and target gene promoters [28]. One such lncRNA is HOX antisense intergenic RNA (HOTAIR), which promotes angiogenesis when upregulated, this activates the transcription of vascular endothelial growth factor by targeting the VEGF promoter [30,31,32]. Additionally, CPS1-IT1 interacts with BRG1 and inhibits the expression of Cyr61 as well as its downstream targets. These downstream targets, namely, VEGF and matrix metalloproteinase 9 (MMP9), regulate tumour angio-genesis [31,33]. Moreover, MMP-9 plays a key role in the remodelling of the extracellular matrix at the onset of neovessel formation. LINC00312 binds to YBX1 and promotes the expression of VEGF while Linc00665 promotes the transcription of ANGPTL4, ANGPTL3 and VEGF through binding to YB-1 [32,33,34,35,36]. Like ANGPTL4, ANGPTL3 regulates EC lipid metabolism and promotes angiogenesis [29]. By modulating proteins that are re-quired in the early stages of tumour vessel formation, these lncRNAs play a key role in the angiogenic switch.

**Figure 3:**
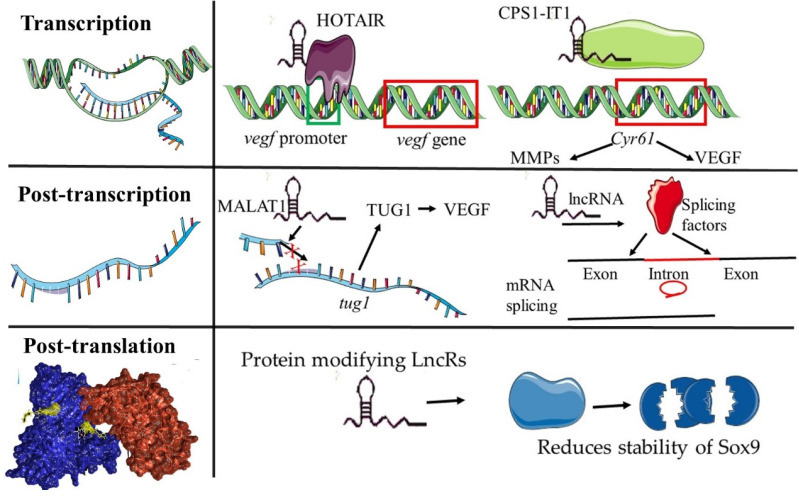
A depiction of the activity of lncRNAs regulating gene transcription in a hierarchical fashion. LncRNAs can control gene expression by regulating the process at different stages. At the level of transcription lncRNAs can recruit transcription factors to promoters or inhibit promoter binding. The Red boxes indicate the target sequence on DNA strands. At the post-transcriptional level, lncRNAs can regulate alternate splicing by associating with splicing factors or altering the degradation of mRNA by regulating the activity of miRNAs. Finally, at the post translational level lncRNAs can modify proteins, for example by reducing the stability of a protein.

At the post-transcriptional level, lncRNAs sequester mi-RNAs, interact with splicing factors and with RNA-binding proteins (RBPs) [37]. MALAT1 and Taurine upregulated gene 1 (TUG1), which are upregulated in various tumours including HCC, colorectal cancer (CRC), breast cancer, glioblastoma, and hepatocellular carcinoma promote angiogenesis by increasing VEGF expression through sponging miRNAs. Microvascular invasion in hepatocellular carcinoma (MVIH) are also overexpressed in HCC and they promote tumour angiogenesis (Figure 3) [38,39,40]. Other mechanistic actions are orchestrated through protein modification and enhancer peptides. Protein-modifying lncRNAs coordinate the activation and stability of some proteins [41,42]. TNK2 Antisense RNA 1 (TNK2-AS1), a protein-modifying lncRNA, promotes angiogenesis via STAT3/VEGF and is upregulated in cancer [43,44]. In contrast, neuroblastoma-associated transcript 1 (NBAT1), which interacts with Sox9 and reduces its protein stability, resulting in anti-angiogenesis [45], is downregulated in gastric cancer (Figure 3) [45,46]. Similarly, the encoding peptide LINC00908 has an anti-angiogenic effect. It encodes ASRPS, which limits STAT3 phosphorylation and thereby inhibits VEGF [47]. LINC00908 is downregulated in triple-negative breast cancer [47].

## 4. Clinical Significance of Angiogenesis Regulating lncRNAs in Cancer

In many cancers the current staging has limitations in terms of determining prognosis. Biomarkers are critical in completing clinical staging and improving the prediction of lymph node metastasis as well as in determining cancer prognosis. Many lncRNAs are over-expressed in various cancer cell lines, as well as in preclinical cancer models and patients. The expression patterns of angiogenesis regulating lncRNAs that have been shown to correlate with disease progression and treatment outcome in cancer patients are listed in Table 1. These lncRNAs have been explored for possible clinical application as biomarkers and as targets for therapeutic intervention [48,49,50,51].

### 4.1. Angiogenesis Regulating lncRNAs: Role as Cancer Biomarkers

Investigations have revealed significant correlations between patient outcome and the expression levels of some of the angiogenesis regulating lncRNAs such as UBE2CP3, LINC00312, and HOTAIR [57,84,87]. In breast cancer, UBE2CP3 is highly expressed, promotes tumour angiogenesis, and is also associated with poor prognosis [18,87]. In patients with HCC, UBE2CP3 expression levels correlated with vessel density. Furthermore, patients overexpressing UBE2CP3 had a median overall survival (OS) that was lower than that of HCC patients with tumours that did not express UBE2CP3 [49]. Interestingly, the expression of UBE2CP3 is restricted to the tumour and has not been detected in the para-tumour tissue [49]. On the other hand, meta-analysis revealed that TUG1, SPRY4-1T1, and HULC did not correlate with lymph node metastasis in various cancers [49,51]. However, a correlation could be established between the expression levels of these three lncRNAs and low overall survival in cancer patients [50].

MVIH was initially identified in hepatocellular carcinoma but was later also detected in other neoplasms such as breast cancer [84,85]. Its overexpression in HCC patients correlates with increased tumour vascularization and metastasis [51,85]. In a mouse model of HCC, the induced overexpression of MVIH stimulated angiogenesis and promoted tumour growth and metastasis [88]. Of note is that, in breast and non-small cell lung cancers, MVIH is associated with poor prognosis, while in HCC patients who undergo hepatectomy it is a predictor of low recurrence free survival [52,88,89,90]. UBE2CP3 and MVIH could thus be useful markers for monitoring patient response to cancer therapy [51,91,92]. HOX transcript anti-sense RNA (HOTAIR), the first antisense transcription lncRNA to be discovered, is overexpressed in cancer tissues compared to normal tissues. It is linked to the development of gastric cancer (GC), breast cancer, lung cancer, and liver cancer [51]. HOTAIR promotes the expression of VEGF and activates the PI3K/AKT/multidrug resistance protein 1 (MRP1) pathway through direct binding to miR-126. Data has shown that serum HOTAIR levels were higher in patients with oesophageal squamous carcinoma when compared to controls without cancer, and that HOTAIR levels correlate with tumour node metastasis (TNM) stage [93]. In gastric cancer, HOTAIR correlates with lymph node and distant metastasis, while in colorectal carcinomas it is associated with advanced stage and metastases [94]. H19, another angiogenesis-regulating lncRNA, is also associated with lymph node metastasis [51]. These lncRNAs could serve as prognostic biomarkers in both gastric and colorectal carcinomas. Moreover, HOTAIR is currently used as a prognostic marker for recurrence in patients who have undergone liver transplantation [95]. Additionally, several studies have shown that blood levels of HOTAIR are good predictors of disease outcome [96]. HOTAIR and PVT1 detected in the saliva of early pancreatic patients were identified as possible biomarkers [97,98,99]. Homebox A11 antisense (HOXA11as) was highly expressed in cancerous tissue, and its expression showed a significant correlation with clinicopathological features in serous ovarian cancer (SOC) [100]. Additionally, patients with an elevated expression of HOXA11as had a significantly shorter progression-free and overall survival rates. These observations provide a basis for the further studies and the development of these lncRNAs as biomarkers.

### 4.2. Therapeutic Targeting of lncRNAs in Cancer Angiogenesis

Several lncRNAs that regulate tumour angiogenesis were shown to be aberrantly expressed in many cancers, making them an attractive target for drug design. Moreover, many of these lncRNAs are not readily detectable in normal tissue, and some are both tissue and cancer subtype specific [51,101]. A few studies have investigated the potential use of these lncRNAs as targets for cancer therapy mainly in preclinical models [65,67,102]. The silencing of SPRYT4-IT1, inhibits the migration of oesophageal squamous cell carcinoma cell in vitro, while NEAT1 suppression inhibits tumour cell growth through p53 [102]. In several independent studies, MALAT1 promoted angiogenesis in vitro and its silencing led to an increase in EC migration, while the inhibition of MALAT1 expression by GapmeR inhibited EC sprout formation [9,65,67]. HOTAIR expression levels were found to be high in cisplatin resistant ovarian cancer cells. The knockdown of HOTAIR in these cells led to the inhibition of tumor cell growth and invasiveness [103]. The silencing of HOTAIR by siRNAs inhibits tumour cell invasiveness in breast cancer and reduces tumour growth in pancreatic cancer [102]. Furthermore, the knockdown of HOTAIR has been shown to improve the sensitivity of tumour cells to cisplatin and doxorubicin. The silencing of CRNDE in colorectal cancer cells suppresses tumour cell growth and reduces resistance to chemotherapy [104]. While these studies have yielded positive results, they are in their infancy and much remains to be done. Additionally, recent reports on the mechanism of some lncRNAs reveal anecdotal results. The LINC000961 gene was shown to yield two molecules with different and opposing effects on angiogenesis [105]. Similarly, another angiogenesis-regulating lncRNA that has been explored for drug targeting is LincRNA-p21 [27]. While some studies showed that it correlates with microvessel density and that its silencing reduces VEGF expression, it was also found to be downregulated in tumour tissue [27]. These findings underscore the importance of more in-depth investigations to elucidate the roles and mechanisms of these lncRNAs.

## 5. Conclusions

**Figure 4:**
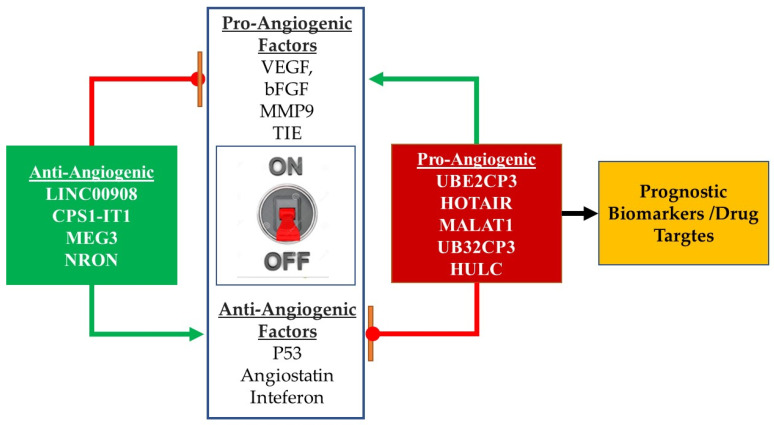
Summary of lncRNAs role in angiogenesis and the practical application of this knowledge. The angiogenic switch relies on the change in the balance between the levels or activity of pro and anti-angiogenic factors. Pro-angiogenic lncRNAs promote the activity of the pro-angiogenic factors while inhibiting the anti-angiogenic factors. The expression profile of these lncRNAs can be used as prognostic biomarkers or as targets for the development of new therapies. Anti-angiogenic lncRNAs promote the activity of anti-angiogenic factors while inhibiting those of the pro angiogenic factors.

It is evident from emerging studies that lncRNAs regulate the fine balance between pro- and antiangiogenic factors, and that their deregulation may contribute to the transition from the dormant avascular tumour to an angiogenic malignant phenotype. Emerging studies have identified several lncRNAs as key regulators of molecules which drive the angiogenic switch, such as VEGF, MMP9, and TIE (Figure 4). While most angiogenesis regulating lncRNAs are upregulated in various cancers, a few of these transcripts which exhibit antiangiogenic activity are downregulated. Of note is that in a diverse array of cancers the expression patterns of these lncRNAs correlate with clinical outcome. The findings of these studies render such angiogenesis modulating transcripts as potential cancer biomarkers. Moreover, some of the lncRNAs are stable in body fluids and can be useful in non-invasive applications. However, future investigations should focus on the sensitivity and specificity of MALAT1 and H19 in cancer detection. Studies with larger samples sizes are required to determine the degree of diagnostic accuracy. Important promoters of tumour angiogenesis can serve as therapeutic targets, including MALAT1, TUG1, LNC00323-003, PVT1, and MIR503HG. Antisense oligonucleotides have been employed for the modulation of gene expression, and their approval for the treatment of patients opens avenues for further exploration in the clinical application of silencing or targeting proangiogenic lncRNAs such as TUG1 and PVT1. The targeting of MALAT1 with GapmeR has recently been shown to be effective in myeloma. There is a need to further elaborate possible drug delivery platforms that will enhance tumour tissue specific targeting to minimize the off-target effects commonly encountered with most anti-cancer treatments. Furthermore, the limitations of current studies on angiogenic lncRNAs is that they have focused on inhibiting vessel formation. On the other hand, it is well-known that tumour blood vessels are structurally and functionally abnormal, and hamper drug delivery. The remodelling of the tumour vasculature may be more advantageous, especially for combination approaches that target various components of the tumour microenvironment, and as such studies are needed to determine ways to optimize lncRNA targeting to normalize tumour vessels and enhance the delivery of chemo- and immunotherapy drugs. In future, technologies such as CRISPR and genome-wide chromatin interrogation will improve our understanding of the functions of angiogenesis regulating lncRNAs and aide in informing drug design approaches.

## Figures and Tables

**Table 1:** Angiogenic LncRNAs with potential as cancer biomarkers.

LncR	Cancer	Expression	Mechanism of Action	Potential Application	Reference
LINC00313	Lung, thyroid	Upregulated	Inhibits the transcription of genes regulating cell motility	Prognosis	[52,53]
CPS1-IT1	Multiple	Upregulated	Inhibits VEGF, MMP-9 and Cyr61	Prognosis	[54,55]
CRNDE	hepatoblastoma, leukemia	Upregulated	Modulates the PI3K/PKB/mTOR pathway	Prognosis, Identification of subtype (in Leukemia)	[56]
HOTAIR	Nasopharyngeal carcinoma	Upregulated	targets the VEGF promoter and activates the transcription of VEGF; modulates Ang2 expression through the upregulation of GRP78	prognosis, recurrence	[57,58,59,60]
HOTAIR	Melanoma	downregulated		prognosis	[59,61]
PVT1	gastric cancer	Upregulated	activates VEGF via STAT3	aggressiveness	[62,63,64]
MALAT1	Multiple	Upregulated	promotes the expression of VEGF, SLUG and Twist	detection, risk of metastasis, prognosis	[65,66]
TUG1	Multiple	Upregulated	modulates HIF-1α expression, promotes VEGF expression	prognosis	[67,68]
LINC00346	Glioma	Upregulated	induces ZNF655 degradation	prognosis	[69,70]
FLANC	CRC	Upregulated	induces VEGF expression via STAT3	prognosis	[71,72]
LINC00908	TNBC HCC	downregulated	inhibits STAT3 phosphorylation, decreases VEGF expression	prognosis	[73,74]
LINC00312	lung cancer Nasopharyngeal carcinoma	Upregulated	induces VEGF expression	prognosis	[48,75]
H19	bladder cancer, gastric cancer	Upregulated	increases VEGF expression	early recurrence, prognosis	[76,77,78,79]
HULC	HCC	Upregulated	promotes SPHKI expression	metastasis	[80,81,82,83]
MVIH	HCC	Upregulated	interacts with PGK1	prognosis	[84,85]
TNK2-AS1	NSCLC	Upregulated		prognosis	[86]
UBE2CP3	glioma, HCC	Upregulated	activates the ERK1/2/HIF-1α/VEGF pathway	prognosis	[49,87]

HCC, hepatocellular carcinoma; CRC, colorectal cancer; NSCLC, non-small cell lung cancer; TNBC, Triple-negative breast cancer; HULC, highly upregulated in liver cancer; HIF, Hypoxia-inducible factor; MMP-9, matrix metalloproteinase-9; PI3K, phosphatidylinositol 3-kinase; PKB, protein kinase B; mTOR, mammalian target of rapamycin; STAT, signal transducer and activator of transcription; GRP, glucose-regulated protein; SPHKI, sphingosine kinase 1; Ang2, angiopoietin2 VEGF, vascular endothelial growth factor; CYR61, Cysteine-rich angiogenic inducer 61; PGKI—Phosphoglycerate kinase; ERK1/2, Extracellular signal-regulated protein kinase 1/2.

## Data Availability

Not applicable.
